# Ultra-Low-Temperature Tensile Fracture Mechanism of 500 MPa Duplex Steel Bar

**DOI:** 10.3390/ma18102288

**Published:** 2025-05-14

**Authors:** Zhenghong Ma, Jun Cao, Huanhuan Zhang, Shubiao Yin, Bingguo Liu, Zhibo Zhang

**Affiliations:** 1 Faculty of Metallurgy and Energy Engineering, Kunming University of Science and Technology, Kunming 650093, China; mzh1070@163.com (Z.M.); jcao_cisri@foxmail.com (J.C.); bingoliu@126.com (B.L.); 2 Jiangsu Yonggang Group Co., Ltd., Suzhou 215000, China; 3 School of Mechanical and Electrical Engineering, Yunnan Open University, Kunming 650500, China; zhangzhibo100@126.com

**Keywords:** low temperature, deformation, ductile fracture, dislocations, duplex microstructure

## Abstract

In the field of low-temperature-resistant steel bars in the liquefied natural gas (LNG) ultra-low-temperature environment, matching the strength and toughness of the material has become a key technical difficulty. In this paper, a duplex low-temperature-resistant steel bar was developed and designed, which adopts a continuous water-penetrating rolling process and a self-tempering process to effectively control the microstructure proportion of it at room temperature and effectively cope with ultra-low-temperature tensile failure at −163 °C. We studied the failure mechanism of 500 MPa steel grade low-temperature-resistant steel bars at tensile temperatures from 25 °C to −163 °C. We define a mixed microstructure of ferrite and pearlite (F + P) as the core of the material and tempered martensitic (TM) as the border of the material. It was found that the core and border microstructure had different response characteristics at different tensile temperatures. It is proved that, through the duplex microstructure design, it can meet the design requirements for the 500 MPa steel grade of low-temperature-resistant steel bars. By clarifying the effects of microstructure deformation, dislocation distribution, precipitated phase, and inclusions on the low-temperature resistance of steel bars under low-temperature tensile fracture, the deformation models of core and border microstructure under different tensile temperatures were constructed, and the methods for optimizing the production process of subsequent steel mills were given. After the optimization, the low-temperature toughness of the 500 MPa steel grade steel bar will be further guaranteed.

## 1. Introduction

Liquefied natural gas (LNG) is a low-carbon, high-efficiency, and high-potential clean fossil energy, which can be used as an important transition energy in the new energy system. In the future, natural gas will account for an increasing proportion of China’s energy microstructure with social development and has broad market development prospects [1,2,3,4]. The LNG storage tank is a high-cap, prestressed concrete cylinder microstructure whose main purpose is to store liquefied natural gas. Because low-temperature-resistant steel bars are mainly used in the reinforced concrete walls and bottom plates of the outer tank of LNG storage tanks, they have a reinforcing and supporting effect on the inner tank of the storage tank and do not directly contact with cryogenic liquids, so the concept is different from that of 9Ni low-temperature steel (steel plate for cryogenic containers), and the performance requirements are also inconsistent [5]. Different types of steel are used at low temperatures, including high-manganese steel [6,7,8], invar steel, 9Ni, 36Ni, aluminum alloy, and austenitic stainless steel [9,10,11,12,13]. These steels are expensive to manufacture, and not all low-temperature steels are used in such demanding scenarios. Most of the use scenarios of low-temperature-resistant steel bars are in low-temperature environments, and it is urgent to develop a steel bar that not only ensures low temperature resistance but also takes into account economic benefits.

**Figure 1:**
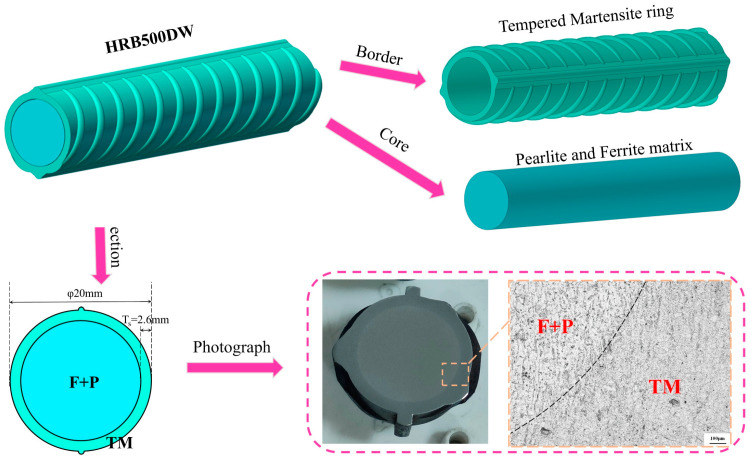
Schematic diagram of the distribution of low-temperature-resistant steel bar.

An important technical indicator of low-temperature-resistant steel is the low-temperature brittleness resistance [14]. Low-temperature brittle fractures mostly belong to cleavage fractures or quasi-cleavage fractures, and the components are in a low-load working state at the time of fracture, the fracture stress does not exceed the yield strength of the material, and the crack propagation rate is fast, which is extremely harmful to building safety. In a certain temperature range, the yield strength and tensile strength of steel increase with the decrease in temperature [15], but the toughness of the material will decrease sharply, and the fracture mode will change from a ductile fracture to a brittle fracture [16,17,18]. When alloy steel is deformed at low temperatures, dislocations tend to accumulate in the grain to a certain extent, and interactions between dislocations, including dislocation absorption, interlacing, and circularization, further increase the dislocation density and significantly affect the mechanical properties of the crystal [19]. Moreover, because the grain boundaries act as weak bonding regions between grains and experience significant stress concentrations, the fracture mechanism gradually changes from a transcrystalline fracture to a intergranular fracture [20]. The alloying element nickel can improve the low-temperature toughness of steel, making it ideal for those who need high resistance to brittle fracture [21], and trace alloying elements, such as Nb and V, can strengthen steel through fine carbonitride precipitation [22]. The advantage of low-temperature-resistant steel bars is that they do not undergo obvious tough–brittle transformation at the set application ambient temperature, and the elongation does not decrease significantly in the low-temperature environment. Before 2013, China’s domestic LNG low-temperature steel bar was mainly imported from Luxembourg Arcelor. In 2014, Maanshan Iron & Steel Company Limited (Maanshan, China)developed a kind of low-temperature steel bar product with mechanical properties at room temperature that met the technical requirements of LNG storage tanks and was applied to the construction site of CNOOC Fujian LNG project [5]. Taiyuan Iron & Steel Group Co., Ltd. (Taiyuan, China) has carried out trial production for steel bar specifications, such as 10~25 mm. At present, there are few studies on the fracture failure mechanism of low-temperature-resistant steel bars in low-temperature environments, and even less research on low-temperature-resistant steel bars of high-strength 500 MPa steel grade. Yan performed low-temperature gradient tensile tests at 335 and 400 MPa, with tensile temperatures ranging from room temperature to −163 °C, with limited strength and elongation after breaking [23]. In order to meet the market demand for low-temperature-resistant steel bars of higher steel grades, we developed and produced 500 MPa steel grade low-temperature-resistant steel bars and studied the failure mechanism of it at different temperatures from a microscopic perspective. The steel bar produced by the steel mill using a continuous water penetration self-tempering process show two concentric cross-sectional layers, a tempered martensitic ring (TM) and a ferrite and pearlite (F + P) core. The properties of the core and border microstructure are quite different, which we define as the duplex structure of low-temperature-resistant steel bars, which is slightly different from the traditional hybrid bidirectional structure, as shown in Figure 1. It is this combination of tempered martensitic with ferrite and pearlite that is the main reason for the high yield strength and good low-temperature toughness of this steel bar.

In this paper, the low-temperature-resistant steel bars of 500 MPa steel grade have been successfully industrialized. We carried out tensile tests at ambient temperatures of 25 °C, −50 °C, −130 °C, and −163 °C; the engineering stress–strain curve was recorded; the tensile fracture was analyzed, and we verified whether the low-temperature-resistant steel bars produced by the enterprise meet the performance requirements of the recommended standard for the ferrous metallurgical industry of the People’s Republic of China [YB/T 4641-2018]. This paper focuses on the analysis of the fracture and failure mechanism of the low-temperature-resistant steel bar in the border TM and core F + P microstructure at different tensile temperatures.

## 2. Test Materials and Methods

All the experimental steel bars are low-temperature-resistant steel bars with a diameter of 20 mm, and the chemical composition is shown in Table 1. First, the matrix of low-temperature-resistant steel bars was preliminarily characterized, including metallographic, matrix hardness, grain size statistics, X-ray diffraction, and transmission. Then, the axial tensile mechanics test under different ambient temperatures was carried out, the tensile ambient temperature was between 25 °C and −163 °C, and the test ambient temperature error could be controlled at ±2 °C. Then, the microscopic analysis of the tensile fractures of the steel bar at different tensile temperatures was carried out, and the differences between the core and border microstructure under low-temperature deformation were compared and analyzed. In order to facilitate readers to quickly understand the microstructure of the test material, we made a schematic diagram of the microstructure distribution of the 500 MPa steel grade low-temperature-resistant steel bar, as shown in Figure 1. The steel bar is φ20 mm, and the thickness of the border TM ring is 2.6 mm.

### 2.1. Preliminary Characterization of Matrix Microstructure

**Figure 2:**
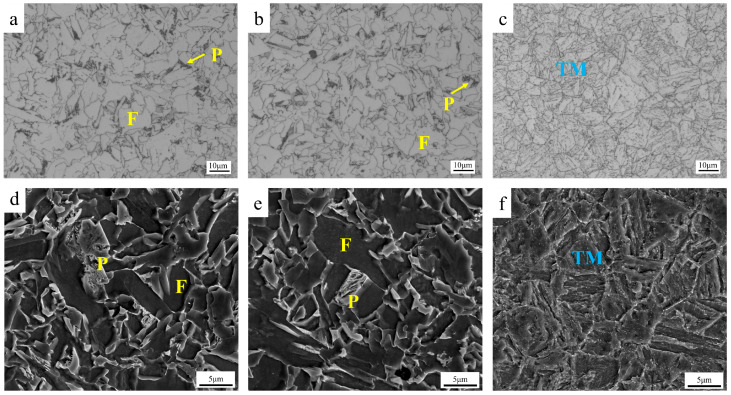
Metallographic microstructure of low-temperature-resistant steel bar. (**a**,**d**) Central microstructure; (**b**,**e**) 1/2 cardiac microstructure; (**c**,**f**) border microstructure.

As shown in Figure 2, the core microstructure of the low-temperature-resistant steel bar is ferrite and pearlite (F + P), and the border microstructure is tempered martensite (TM). TM microstructure stiffness is higher than that of F + P microstructure. The average Vickers hardness of the core microstructure was 212HV_0.3_, and the hardness of the border microstructure was 293HV_0.3_. According to the grain size statistics of low-temperature-resistant steel bar, the average grain size of the core is 4.76 μm, and the average grain size of the border is 6.05 μm. The preliminary characterizations are summarized in Table 2.

### 2.2. X-Ray Diffraction Analysis of Matrix Microstructure

**Figure 3:**
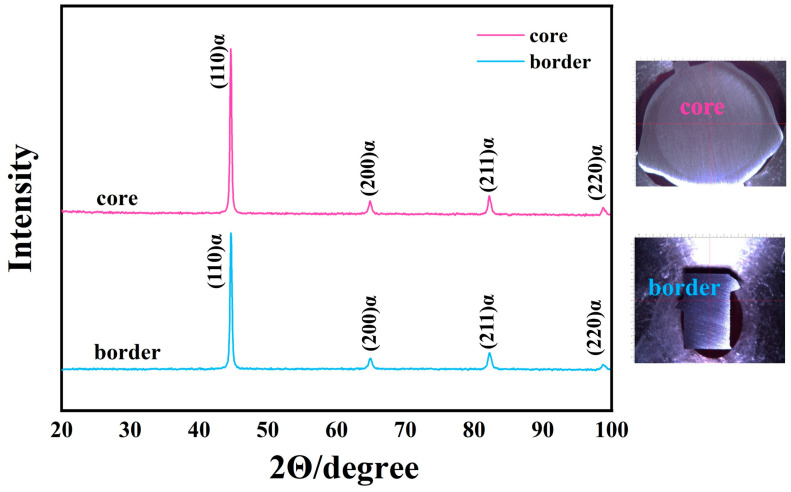
X-ray diffraction analysis results of low-temperature-resistant steel bar.

X-ray diffraction technology (XRD-6100, Japan) was used to analyze the phase difference between the matrix microstructure in the core and the border, the center of the cross-section of the rebar sample was selected for the X-ray scanning area of the core, and the rib area of the longitudinal section of the rebar was selected for the border microstructure, so as to ensure the accuracy of sampling. According to the analysis results in Figure 3, the XRD spectrum shows four absorption peaks, which are (110) α, (200) α, (211) α, and (220) α, and the corresponding 2θ are 44.3°, 64.4°, 82.1°, and 100.4°, which confirms that the phase of the core and border of the low-temperature-resistant rebar is mainly composed of the α-Fe phase. The phase composition of iron–nickel, iron–manganese, and iron–vanadium compounds was detected; the atomic radius difference between iron–nickel and nickel was very small, and it was infinitely soluble [24].

### 2.3. Transmission of Lamellae from the Matrix Microstructure

**Figure 4:**
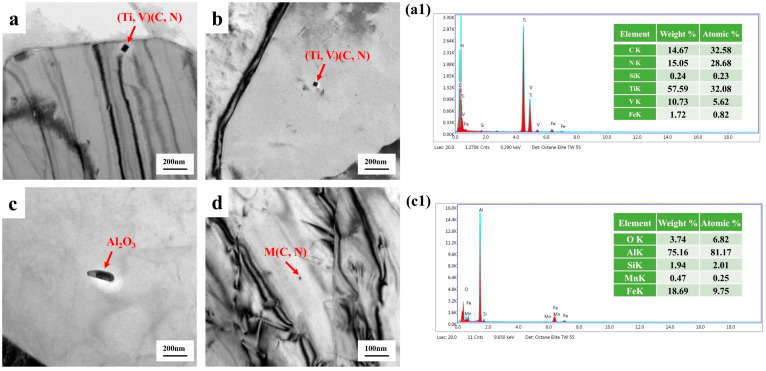
Transmission characterization of low-temperature-resistant steel bar (core). (**a**,**b**) precipitated phase of (Ti, V)(C, N), (**c**) precipitated phase of (Al_2_O_3_), (**d**) precipitated phase of Mn (C, N), (**a1**) energy spectrum of (Ti, V)(C, N), (**c1**) energy spectrum of (Al_2_O_3_).

**Figure 5:**
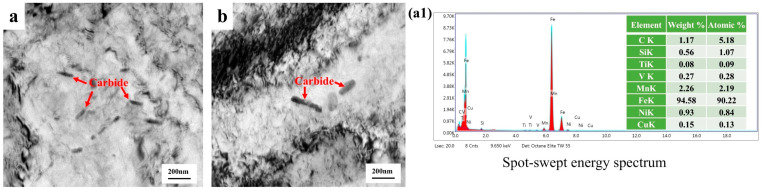
Transmission characterization of low-temperature-resistant steel bar (border). (**a**,**b**) precipitated phase of Carbide, (**a1**) energy spectrum of Carbide.

In the preliminary characterization of the low-temperature-resistant steel matrix, we observed its second-phase precipitated particles. As shown in Figure 4, the core F + P microstructure underwent a self-tempering process, and (Ti, V) (C, N) particles with a diameter of 20–40 nm and some particles below 10 nm appear in the matrix, so it is difficult for the projection device to effectively analyze the energy spectrum due to the small size of the precipitated particles. Nanoprecipitates play a crucial role as reinforcing agents, especially in terms of improving ductility and altering deformation mechanisms. Nanoprecipitates can increase strength without sacrificing elongation [25]. The core matrix also has short rod-shaped Al_2_O_3_ inclusions, which are brittle inclusions of high hardness and are harmful to the quality and life of the material. Figure 5 is the projection characterization of the border TM microstructure, which is not as abundant as the core due to the rapid cooling phase transition of the border microstructure due to the water penetration, and we only observe short rod carbides in the 100–300 nm length.

### 2.4. Test Methods

At the ambient temperature of 25 °C, −50 °C, −130 °C, and −163 °C, the axial tensile test of the low-temperature-resistant steel bar was carried out, and the fracture of the steel bar was cut. Low-temperature tensile fractures will condense moisture in the air and freeze, and fractures are prone to rust. To treat the fracture rust, it is necessary to immerse the rust with 7.5% concentration of hydrochloric acid alcohol in the solution for 1 s; a large number of bubbles will be generated on the surface of the steel bar fracture immediately, and the rust point will be basically removed. Later, under the electron microscope scan, the fracture morphology was photographed, and the smooth shape of the mushroom was observed, which was left after the rust was removed. It is worth noting that the fracture rust should not be too serious, otherwise the fracture topography will be covered by the mushroom-like topography, and the fracture analysis will be meaningless. After the fracture morphology is photographed, the middle deformation section of the fracture specimen is cut off and cut from the center along the axis direction to prepare the metallographic specimen. The surface of the specimen was ground and polished, and the sample surface was corroded with a 4% vol fraction of nitric acid alcohol solution. Then, the deformed microstructure of the test specimen was observed with a GX51 optical microscope (China) and a quanta650 scanning electron microscope (USA). Transmission Electron Microscope (Japan) and electron backscatter diffraction (Japan) analyses were carried out on the fracture necking part, and sub-ion polishing or vibration polishing was recommended for electron backscatter sample preparation to avoid the situation that the resolution of the sample surface was not high due to excessive internal stress. The core and border microstructure of the necking part of the steel bar fracture were observed, and the characteristics of the microstructure at different fracture temperatures were analyzed.

## 3. Test Results

### 3.1. Mechanical Properties

The surface quality of the low-temperature-resistant steel bar was evaluated by bending it forward and backward at room temperature with a diameter of 20 mm. Low-temperature axial tensile tests at 25 °C, −50 °C, −130 °C, and −163 °C were carried out to verify whether the performance of it met the design requirements. The engineering stress–strain curve is recorded throughout the low-temperature tensile process, and the tensile ambient temperature is maintained at the design temperature value ±2 °C.

#### 3.1.1. Bending Test

**Figure 6:**
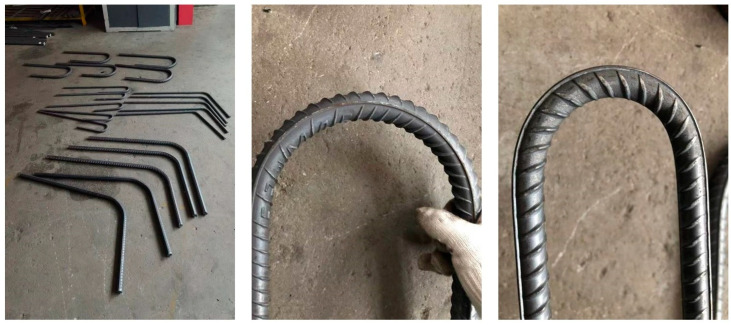
Low-temperature-resistant steel bar forward and reverse bending site.

According to the recommended standard of ferrous metallurgy industry of the People’s Republic of China [YB/T 4641-2018], the forward and reverse bending test of low-temperature-resistant steel bars is carried out. After the positive bending is bent 180° according to the specified bending head diameter, no cracks shall occur on the surface of the bending part of the steel bar. The core diameter of the reverse bend test is correspondingly increased by one bar diameter compared to the bending test. In the reverse bending test, the forward bending is 90°, and then, the reverse bending is 20°. After the reverse bending test, no cracks shall occur on the surface of the bending part of the steel bar. As shown in Figure 6 and Table 3, the test results of the forward and reverse bending tests of low-temperature-resistant steel bar are all of acceptable quality.

#### 3.1.2. Axial Tensile Test

**Figure 7:**
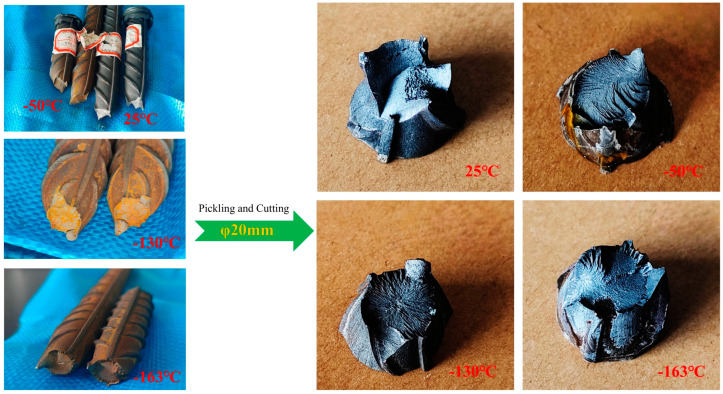
Fracture specimens at different tensile temperatures.

In this paper, the tensile test of low-temperature-resistant steel bars is carried out at four ambient temperatures, namely 25 °C, −50 °C, −130 °C, and −163 °C, where −163 °C is the temperature at which the LNG storage tank stores LNG. The surface of the steel bar in the low-temperature tensile test will be frozen, the tensile fracture is easy to rust, and the fracture observation must be pickled. The tensile fracture is shown in Figure 7. R_eL_ is used as the lower yield strength of the material, Rm is the tensile strength of the material, and the elongation A% after fracture is an important index of low-temperature-resistant steel bars [26,27].

**Figure 8:**
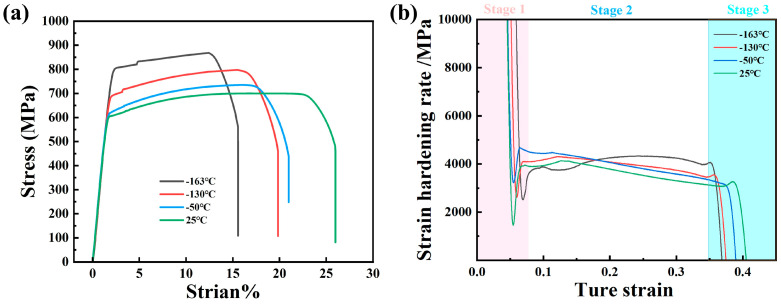
(**a**) Engineering stress–strain curve; (**b**) strain-hardening rate curve.

The engineering stress–strain curve in Figure 8a shows that the tensile strength at room temperature 25 °C is 697 MPa, and the elongation after fracture is 26%. The tensile strength at −163 °C is 873 MPa, and the elongation after breaking is 16%. The strain-hardening rate in Figure 8b shows that stage 1 is the linear elastic deformation stage, stage 2 is the molding deformation stage, and stage 3 is the tensile necking stage. The strain-hardening rate represents the stress increment corresponding to the unit strain increment, which characterizes the difficulty of the material to continue plastic deformation. In order to facilitate the comparison of the change in the strain-hardening rate at various temperatures, we do not exclude the elastic strain stage, and the real scope of the strain-hardening rate is defined from the shaping deformation in the second stage. The lower the tensile temperature of the low-temperature-resistant steel bar, the slower it will enter the molding deformation in stage 2. When entering the molding deformation in stage 2, the lower the tensile temperature, the higher the strain-hardening rate is maintained, and the work-hardening effect of −163 °C is significantly stronger than that of 25 °C [28,29]. The following is the formula for calculating the strain-hardening rate of the material. The results are shown in Figure 8b.

The engineering stress is *σ_eng_*, the engineering strain is *ε_eng_*, A_0_ is the original cross-sectional area of the specimen, and L_0_ is the original gauge length of the specimen, as follows.

(1) $$\sigma_{eng}=\frac{F}{A_{0}}\;\varepsilon_{eng}=\frac{\Delta L}{L_{0}}$$

True stress is *σ_true_*, and true strain is *ε_true_*, as follows:

(2) $$\sigma_{true}=\sigma_{eng}\left(1+\varepsilon_{eng}\right),\;\varepsilon_{true}=\ln\left(1+\varepsilon_{eng}\right)\;$$

The strain-hardening rate is defined as the derivative of true stress (*σ_true_*) to plastic strain (*ε_p_*), as follows:

(3) $$Strain\;hardening\;rate=\frac{d\sigma_{true}}{{d\varepsilon }_{true}}$$

In the low-temperature environment, the pinning effect of impurity atoms (such as carbon and nitrogen) or precipitated phases on the dislocation is enhanced at low temperatures, which hinders the dislocation slip [27,30], which in turn reflects the increase in the tensile strength of the material at low temperatures. When the plastic deformation cannot relieve the stress, the material may fail rapidly along the (001) crystal plane, resulting in more brittle fracture modes in the low-temperature tensile fracture. The tensile strength of ordinary steel at a low temperature will also increase, but its elongation will decrease significantly with the decrease in tensile temperature, which is the tough–brittle transformation of the material in a low-temperature environment [16,31]. The tensile strength of the 500 MPa steel grade studied in this paper increases with the decrease in tensile temperature, and the elongation A% after the fracture and elongation of maximum force A_gt_% decreases slightly. It shows that the low-temperature-resistant steel bar developed in this paper can resist the damage of reduced toughness at low temperatures, and the obvious tough–brittle transformation will not occur, even at −163 °C. Table 4 and Table 5 below are the performance requirements for low-temperature-resistant steel bars in the recommended standard YB/T 4641-2018 for the ferrous metallurgical industry of the People’s Republic of China. Table 6 shows the results of the low-temperature axial tensile test of low-temperature-resistant steel bar.

### 3.2. Tensile Fracture Microstructure Analysis

#### 3.2.1. Fracture Profile Microstructure

**Figure 9:**
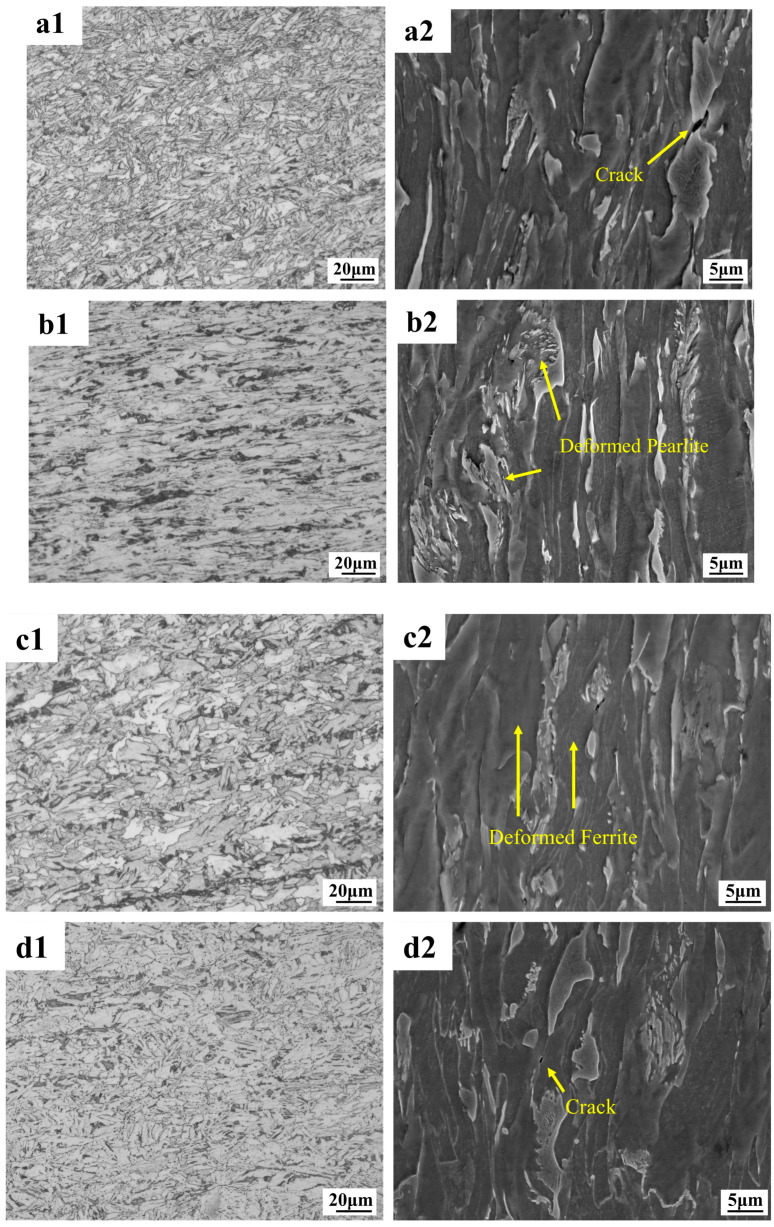
Tensile fracture profile of low-temperature-resistant steel bar (core). (**a1**,**a2**) 25 °C; (**b1**,**b2**) −50 °C; (**c1**,**c2**) −130 °C; (**d1**,**d2**) −163 °C.

**Figure 10:**
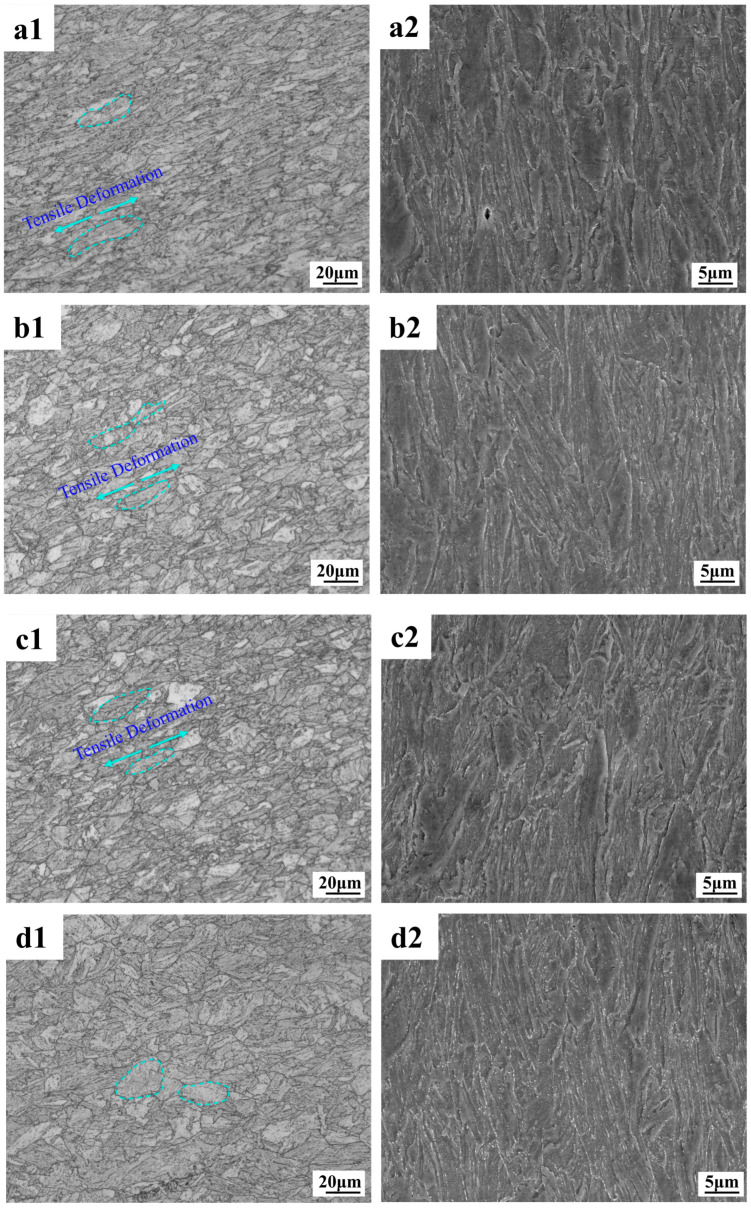
Tensile fracture profile of low-temperature-resistant steel bar (border). (**a1**,**a2**) 25 °C; (**b1**,**b2**) −50 °C; (**c1**,**c2**) −130 °C; (**d1**,**d2**) −163 °C.

As can be seen from the metallographic and electron microscope scans in Figure 9, the deformation of the F + P microstructure in the core is more intense, and cavities and cracks are prone to appear around the pearlite microstructure. Because the lamellar cementite in pearlite is harder than ferrite, this area is prone to stress concentrations that cause cavities and cracks to appear first. As shown in Figure 10, the border microstructure is relatively uniform, and the probability of cavities and gaps between different grains is lower than that of the core microstructure. From the perspective of the overall degree of deformation, the deformation of the core is higher than that of the border microstructure. Interestingly, under the tensile fracture of −163 °C, the overall deformation degree of the core microstructure is similar to that of other tensile temperatures, but the overall deformation degree of the border microstructure Figure 10(d1,d2) is significantly smaller than that of other tensile temperatures. This shows that the F and P microstructure of the core of the low-temperature-resistant steel bar have good overall deformation ability at different temperatures, while the overall coordinated deformation ability of the border TM microstructure will be significantly reduced at low temperatures.

#### 3.2.2. Fracture Profile EBSD

The data in Table 7 are the statistics of the block size parameters, the proportion of grain boundaries, and the degree of the dislocation density accumulation of grain before and after the tensile of the low-temperature-resistant steel bar. It should be noted that AZtecCrystal (2023) automatically counts a large number of subgrain boundaries and precipitated phases, which leads to a small overall average size of the grain block. The grain sizes we usually discuss are based on the data in Table 2 above. Table 7 shows the overall deformation trend of the grain block at different tensile temperatures.

**Figure 11:**
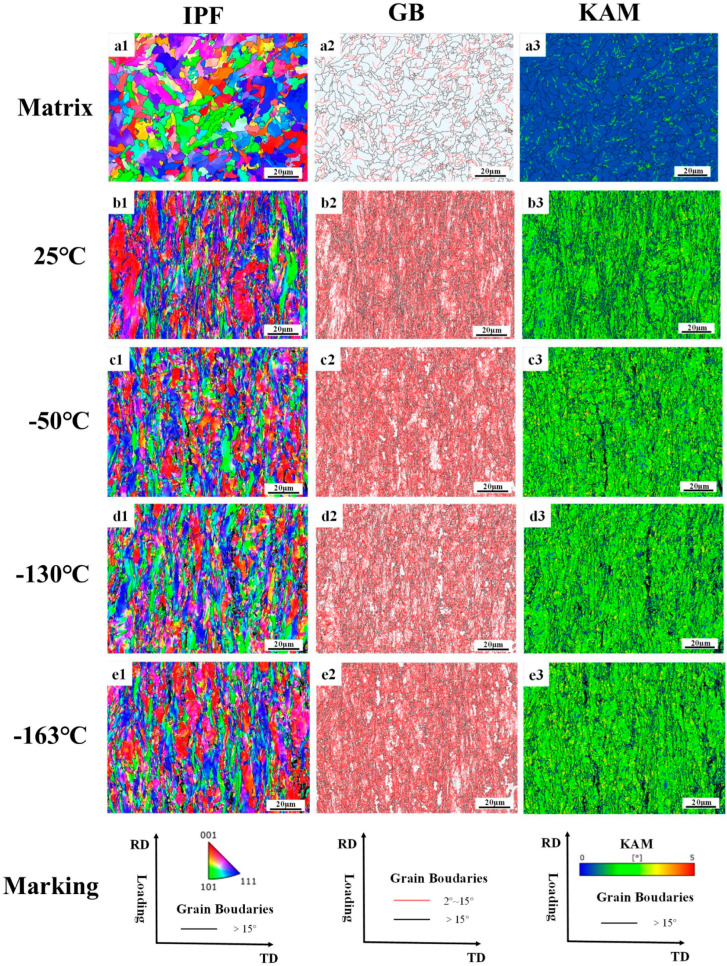
Electron backscatter analysis of tensile fractures of low-temperature-resistant steel bar (core). (**a1**–**a3**) Martix, (**b1**–**b3**) 25 °C, (**c1**–**c3**) −50 °C, (**d1**–**d3**) −130°C, (**e1**–**e3**) −163°C.

Figure 11 shows the electron backscatter results of F + P in the core of the rebar. The GB diagram shows the size and angle grain boundaries of the matrix at the core of the rebar, with large angle boundaries defined as larger than 15° and small angle grain boundaries defined less than 15°. Most of the undeformed grain boundaries are large-angle grain boundaries, and only a few small angle grain boundaries are present. After tensile deformation, the large-angle grain boundaries undergo a violent extension motion, and the small-angle grain boundaries increase suddenly. The grain boundaries experience a strong dislocation slip movement [32]. Dislocations are line defects in crystals whose motion dominates the plastic deformation of metals. The simple aggregation of dislocations does not directly form grain boundaries but may form subgrain boundaries [33]. The increase in the interaction between dislocations and grain boundaries leads to the formation of low-angle grain boundaries, which gradually accumulate and evolve into high-angle grain boundaries, ultimately contributing to the formation of finer grain boundaries [34,35]. Interestingly, there are some large black areas in the KAM diagram under the tensile deformation fracture, which is actually the place where the dislocation slip aggregation is most severe. Because the deformation is too severe, the lattice distortion of the microstructure here is serious, which leads to the failure of the electronic backscatter signal detection system. Comparing the fracture microstructure of the core at different tensile temperatures, it can be found that the deformation of the core microstructure at −163 °C has the most areas that cannot be resolved by the system, because the matrix of the core undergoes a more intense dislocation slip accumulation at −163 °C. As shown in Table 7, the proportion of large-angle grain boundaries and small-angle grain boundaries in the core fractures at different tensile temperatures is similar, there is no fluctuation after low-temperature stretching, there is no large fluctuation in the KAM dislocation density statistics in the core, and the grain size after fracture is also relatively close. It can be concluded that the deformation ability of the core F + P microstructure is weakly affected by the ambient temperature, which is the reason to ensure that the low-temperature-resistant steel bar still maintains a certain plasticity in the low-temperature tensile environment.

**Figure 12:**
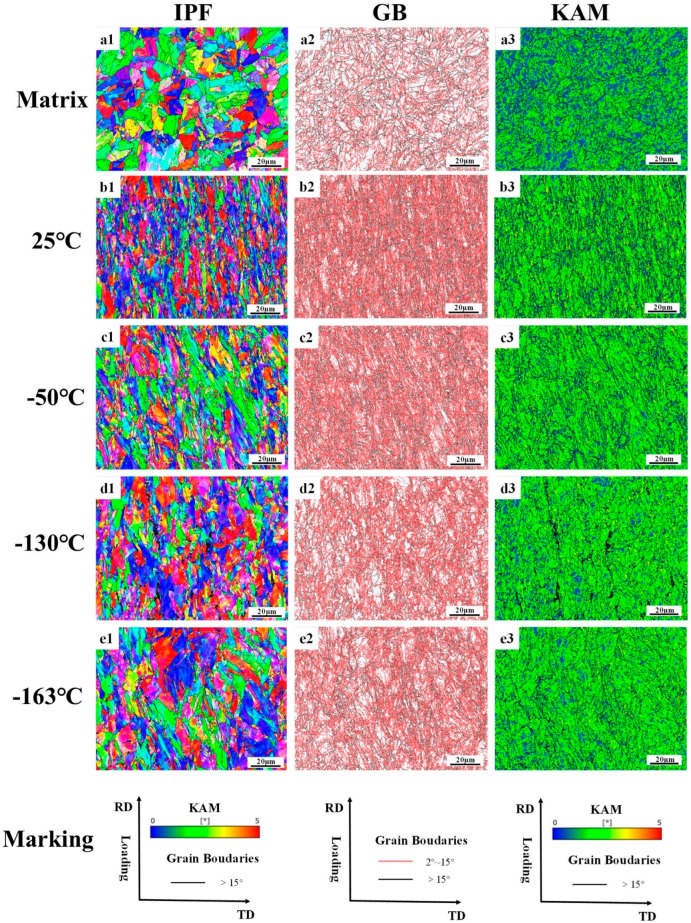
Electron backscatter analysis of tensile fractures of low-temperature-resistant steel bar (border). (**a1**–**a3**) Martix, (**b1**–**b3**) 25 °C, (**c1**–**c3**) −50 °C, (**d1**–**d3**) −130 °C, (**e1**–**e3**) −163°C.

Figure 12 shows the results of the electron backscattering of tempered martensitic in a rebar border matrix. Tempered martensite is the BCC phase. The large number of small-angle grains in the undeformed TM microstructure is due to the martensite’s own lath layer microstructure and dislocations, which work together to divide the matrix, and the electron backscattering system determines that it is a subgrain boundary [36]. Residual stresses develop macroscopically within the material, their length scale is much larger than the grain size, and the volume expansion of the martensitic phase transition will produce a large number of residual stresses on the surface of the material [37]. The TM microstructure without tensile deformation has a high residual stress at room temperature, while the core F + P only has a small amount of residual stress at the grain boundary. This is illustrated by KAM diagram a in Figure 10. Comparative analysis of the border fracture microstructure under different tensile temperatures shows that the overall deformation of the border fracture at −163 °C is the weakest, and the stress accumulation is the most obvious at −163 °C fracture. Therefore, the lower the tensile temperature, the more difficult it is for the TM microstructure to deform, which is different from the case of F + P in the core. As shown in Table 7, the grain size of the −163 °C border fracture is similar to that of the undeformed border microstructure, and the dislocation accumulation of the −163 °C fracture is stronger than that of the 25 °C fracture. This indicates that the border TM microstructure is greatly affected by the change in tensile temperature. The TM microstructure in the low-temperature environment is less deformed than the F + P microstructure in the core. These laws correspond to the conclusions drawn in Figure 7 and Figure 8 above. The core microstructure of the steel bar mainly ensures the toughness of the material in the low-temperature environment, while the border microstructure can ensure that the overall strength grade of the steel bar exceeds 500 MPa.

### 3.3. Tensile Fractures Analysis

Generally speaking, the fracture mode of a material can be judged by observing the microscopic morphology of the fracture. Due to the tensile in the liquid nitrogen environment, frost will inevitably form on the surface of the sample, which will lead to rust on the fracture, and it is necessary to pay attention to the rust of the fracture, as it should not be too severe, otherwise the rust will cover the dimple and cleavage plane morphology, resulting in analysis errors. Ductile fractures are characterized by obvious macroscopic plastic deformation and a large number of dimples at the microscopic part of the fracture. Brittle fractures are characterized by almost no plastic deformation, and fractures develop along grain boundaries called cleavage planes. There is also a quasi-cleavage fracture, which is between brittle fracture and ductile fracture, and the fracture microstructure contains both the dimple under ductile fracture mode and the cleavage surface under brittle fracture mode [38,39]. Most materials will have the characteristics of ductile fracture and brittle fracture at the same time when fractured in a low-temperature environment, so we can judge that the fracture mode of the material is more biased by counting the proportion of fracture dimples and cleavage surfaces. In addition to the fracture dimple morphology, we also analyzed the microscopic changes in the core and border microstructure and elucidated the characteristics of the deformation of the microstructure at different temperatures at the nanoscale.

#### 3.3.1. Topography Analysis of Tensile Fractures

**Figure 13:**
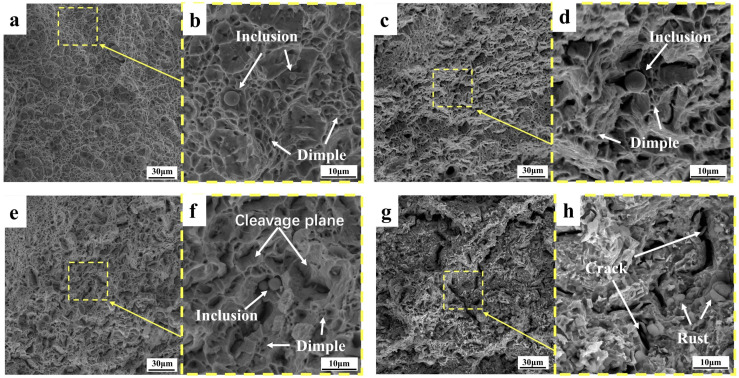
Tensile fracture of low-temperature-resistant steel bar (core). (**a**,**b**) 25 °C; (**c**,**d**) −50 °C; (**e**,**f**) −130 °C; (**g**,**h**) −163 °C.

**Figure 14:**
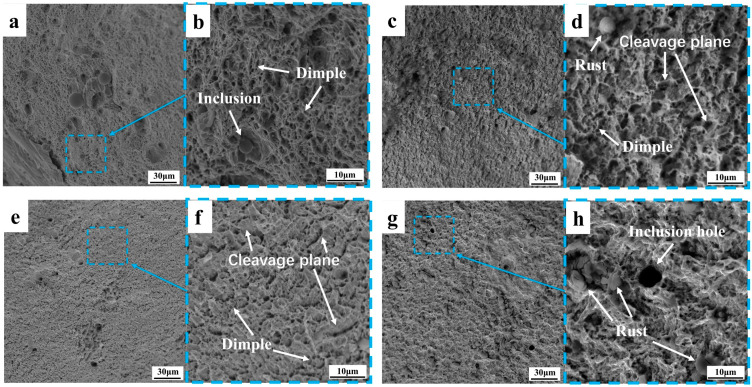
Tensile fracture of low-temperature-resistant steel bar (border). (**a**,**b**) 25 °C; (**c**,**d**) −50 °C; (**e**,**f**) −130 °C; (**g**,**h**) −163 °C.

The fracture morphology of the core of the rebar at different tensile temperatures is shown in Figure 13. With the decrease in tensile deformation temperature, the probability of cleavage surface increases, and the size and proportion of dimples gradually decrease. At 25 °C, a fracture exhibits full dimple fracture form, with a number of large and deep pit. At −50 °C, a fracture exhibits full dimple fracture form, with a few large and deep pit. The cleavage plane of the core at −130 °C accounted for significantly more than that of the core fracture at −50 °C, and the dimple and quasi-cleavage plane were intermingled. At the core fracture at −163 °C, the fracture dimple accounted for a small proportion, and the crack penetrating the matrix was found for the first time. The fracture morphology of the border of the rebar at different temperatures is shown in Figure 14, and the macroscopic morphology of the fracture is seen under the scanning electron microscope enhanced by 1000 times. The fracture morphology at different tensile temperatures is relatively flat, and the size of the dimple and cleavage surface at the border fracture are uniform, unlike the size of the dimple and cleavage surface at the core fracture. With the decrease in temperature, the proportion of the dimple decreases and the proportion of cleavage surface increases, but the proportion of the dimple decreases less than that of the core. No cracks were found penetrating the matrix in the border fracture at −163 °C, and there is still a considerable amount of fracture dimples in the fracture. The core microstructure is F + P, and the strength and hardness of the two microstructures are different, resulting in the stress concentration of the pearlite when stressed at a low temperature, while there is only one TM microstructure at the border, which is relatively uniform.

As shown in Figures 13d,f and Figure 14b, non-deforming inclusions were found at the fracture, and the particle size of the regular spherical inclusions was about 1–10 μm, which were distributed in the deep dimples on the matrix and were judged to be silicate inclusions, mainly calcium silicate, alumina, and sulfide composite inclusions. This kind of spherical inclusion is harmful to the performance of steel bars, because such inclusions have high stiffness and are not easy to deform. Stress concentration first occurs when the steel bar is stressed, and the inclusions form cavities and separate from the matrix, reducing the force uniformity of the material [40]. The smelting process requires control of the content and size of this inclusion.

With the decrease in tensile temperature, the proportion of fractures in the form of a brittle fracture increases, but the tensile fracture at −163 °C is not a complete brittle fracture mode but a quasi-cleavage fracture. The deformation-induced dislocations mainly slide along the same slip system until a critical accumulation threshold is reached, where cracks begin to form [41]. During tensile deformation, many immovable dislocations are formed, which create obstacles in the material, causing stresses to concentrate around these dislocations. This stress concentration significantly reduces the plastic work consumed by the material per unit area, allowing small cracks to propagate quickly and form larger cracks. This effect is even more pronounced at low-temperature tensile conditions, to the point that a large number of cracks appear in the core fracture at −163 °C. Combined with the elongation of low-temperature-resistant steel bar, the elongation after break at the tensile temperature of −163 °C decreases slightly, and the elongation after break is still quite rich compared with the requirements of YB/T 4641-2018. Therefore, the quasi-cleavage fracture form of −163 °C is an acceptable tensile fracture form, which proves that the low-temperature-resistant steel bar has good low-temperature toughness.

#### 3.3.2. Transmission Electron Microscopy Analysis

During the deformation process, the stacked dislocations underwent a dynamic recovery, which was rearranged by slipping and climbing to form a more regular dislocation wall. When the steel undergoes plastic deformation, the dislocation density increases significantly, and the dislocations are intersected and entangled with each other, forming a high-density dislocation network. The dislocations are gradually rearranged, forming a cell-like microstructure of low-density dislocation regions (intracellular) and high-density dislocation walls (cell walls). At this point, the cell wall can be regarded as a subgrain boundary (small-angle grain boundary), and its orientation difference is usually less than 15°, but the grain boundary in the traditional sense has not yet been formed. In order to reduce the internal energy of the system, high-density dislocations will annihilate and rearrange near the dislocation entanglement and dislocation wall, and the disordered dislocation microstructure will gradually evolve into dislocation cells to balance the energy of the system [42,43,44].

**Figure 15:**
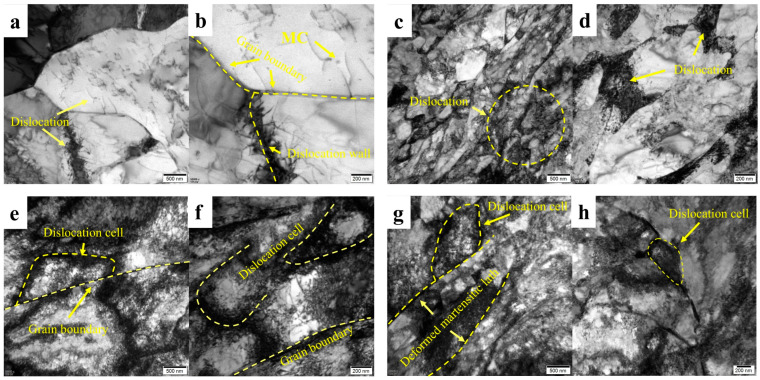
Transmission of fracture microstructure of low-temperature-resistant steel bar (core). (**a**,**b**) Matrix; (**c**,**d**) 25 °C; (**e**,**f**) −163 °C fracture profile direction; (**g**,**h**) −163 °C fracture parallel planes.

As shown in Figure 15a,b, the core microstructure is not tensile and deformed, only some dislocations exist in the grain boundaries, and there are few dislocations in the matrix. Figure 15c–h is a sample that passes through the vicinity of the tensile fracture. The comparison shows that the distribution of dislocations in the core microstructure is different at different tensile temperatures. As shown in Figure 15c,d, the dislocation distribution of the 25 °C fracture is relatively uniform, no complete dislocation cells are formed, and the dislocation accumulation at the grain boundary is not particularly obvious. On the fracture Figure 15e,h at −163 °C, we studied the longitudinal and transverse samples of the tensile fracture; regardless of the direction, the dislocations were heavily aggregated at the grain boundaries, and the dislocations were continuously intersected to divide the matrix and form dislocation cells. At the same time, the dislocation at the −163 °C core fracture extended from the grain boundary to the matrix, and a large number of dislocation cells were formed by continuous intersection.

In the F + P mixed microstructure of the core, the dislocation will react differently at different tensile temperatures. At room temperature, dislocation can easily interact with dislocations in the opposite direction, leading to annihilation and microstructure restoration [45,46,47,48]. However, at low temperatures, the dynamic recovery of dislocations is inhibited, leading to dislocation entanglement and the formation of dislocation cells [49,50]. In the low-temperature environment, the dislocation movement is blocked, the BCC microstructure has fewer slip systems, and the dislocation slip at a low temperature requires higher energy [51]. At the same time, the thermal vibration of atoms is weakened, the amplitude of atomic vibrations is reduced, and it is difficult for dislocations to overcome lattice resistance with the help of thermal activation, resulting in a decrease in plastic deformation ability [41]. This also explains that the tensile strength is greatly increased at low-temperature stretching, while the plasticity decreases. However, at the tensile temperature of 25 to −163 °C, the low-temperature-resistant steel bar always maintains a certain plastic deformation capacity, and even at −163 °C, it does not reach complete brittle failure.

**Figure 16:**
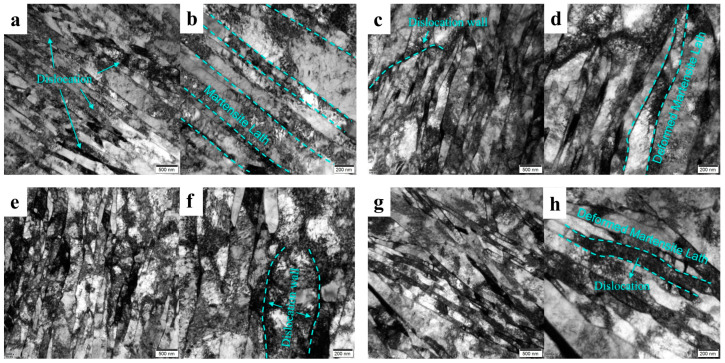
Transmission of fracture microstructure of low-temperature-resistant steel bar (border). (**a**,**b**) Matrix; (**c**,**d**) 25 °C; (**e**,**f**) −163 °C fracture profile direction; (**g**,**h**) −163 °C fracture parallel planes.

Figure 16 shows the transmission microstructure of the fracture border of the low-temperature-resistant steel bar at different deformation temperatures, which is mainly composed of martensite lath, with a slat width between 200 and 600 nm, and the martensite slat width remains relatively consistent. This lack of change is due to the nature of the martensite phase transformation, which is formed by the non-diffusion, shear-dominated transformation of the parent austenite, the basic microstructure of Fe-C alloys [52]. At the same time, the atoms move collectively in a coordinated manner, reorganizing the crystal lattice, which is driven entirely by the difference in chemical potential before and after the transformation. The aligned slatted martensitic exhibits itself through a relatively low shear stress-induced slip mechanism, resulting in some dislocations within the matrix [53]. Figure 16a,b is the martensitic lath that have not undergone tensile deformation, and it can be seen that the lath boundaries are still relatively regular, the lath boundaries tend to be parallel to each other, and there are some dislocations between the lath boundaries [52]. Figure 16c–h is deformed by tensile deformation, and it is clear that the martensitic lath microstructure is bent and deformed, and dislocation accumulation is more pronounced within the basal body. Comparing the results of Figure 16c–f shows that the lath boundary is tortuous and sharp at the −163 °C deformation temperature, and the dislocations are more obvious along the lath boundary. At the fracture of −163 °C, the longitudinal and transverse microscopic deformation morphology is also different. Figure 16e,f is the longitudinal morphology, and Figure 16g,h is transverse, because the observed area is in the area of steel bar necking. This region is accompanied by the centripetal contraction trend, resulting in a complex and strong transverse deformation degree in this area. As shown in Figure 16g, the martensitic laths cross and cover each other, resulting in a high dislocation density in the transverse cross-section. It can be concluded that the hardness of the border TM microstructure is higher than that of the core F + P at room temperature, and a certain number of dislocations accumulate before it is deformed. Therefore, at the lower the tensile temperature, we believe that the increase in the anti-deformation ability of the border microstructure is not as much as that of the core microstructure; that is, the core microstructure is more sensitive to low temperatures.

## 4. Discussion

We discuss the influence of tensile deformation temperature on mechanical properties, the influence of tensile deformation temperature on microstructure, and the advantages of duplex microstructure design in a low-temperature deformation environment. The low-temperature failure model of the core and border microstructure was constructed, the differences between the core and border microstructure affected by low temperatures were emphatically distinguished, and optimization suggestions were given.

### 4.1. Effect of Tensile Deformation Temperature on Mechanical Properties

**Figure 17:**
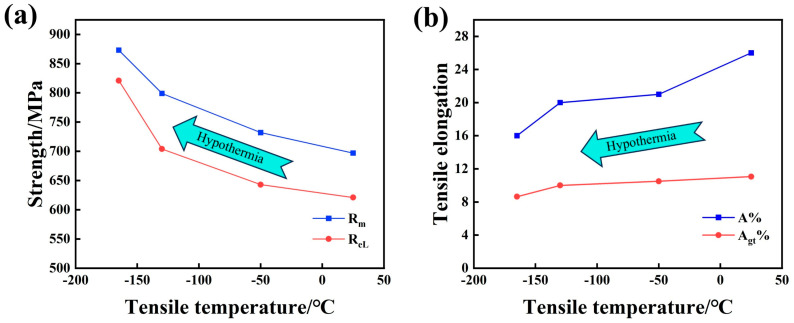
Low-temperature tensile properties of 500 MPa low-temperature-resistant steel bar. (**a**) Yield strength and tensile strength; (**b**) elongation and maximum force elongation.

As shown in Figure 17, the lower the tensile temperature, the higher the strength of the low-temperature-resistant steel bar, and the elongation after fracture decreases [23,29,54,55,56,57,58]. The yield strength at −163 °C tensile temperature is 821 MPa, the tensile strength is 873 MPa, the elongation after fracture is 16%, and the maximum force elongation is 8.64%. Compared with the tensile temperature of 25 °C, the yield strength is increased by 32.2%, the tensile strength is increased by 25.3%, the elongation after fracture is reduced by 38.4%, and the maximum force elongation is reduced by 21.9%. With the decrease in deformation temperature, the vibration of atoms decelerates, the difficulty of atomic motion increases, and the dynamic recovery of dislocations is inhibited, resulting in dislocation entanglement and accumulation, and dislocation strengthening dominates at low temperatures [29,58,59,60]. With a higher strain-hardening rate, tensile strength increases, and plastic deformation ability decreases.

### 4.2. Effect of Tensile Deformation Temperature on Microstructure Deformation

**Figure 18:**
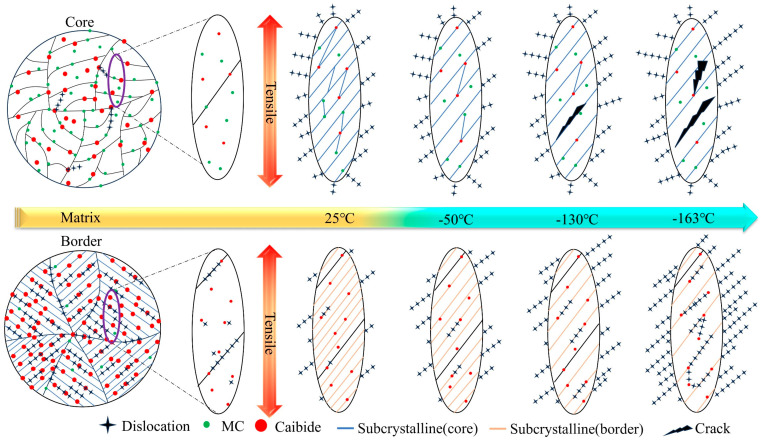
Core and border deformation models vary with different temperatures.

Based on the abovementioned microscopic analysis of fracture at different tensile temperatures, we summarize the deformation laws of core and border microstructure and describe dislocations, precipitated phases, carbides, grain boundaries, and subgrain boundaries in the material matrix. In order to facilitate readers to understand our work more clearly and quickly, we make the model of the deformation of the core and border microstructure of the low-temperature-resistant steel bar in Figure 18 at different tensile temperatures.

We first discuss the core microstructure. The deformation resistance of the F microstructure is less affected by low temperatures, and it shows relatively good deformation ability and fracture toughness after experiencing an ambient tensile temperature of 25 °C to −163 °C [61]. The deformation resistance of P microstructure in the core is more affected by ultra-low temperatures than that of the F microstructure. At the ultra-low temperature of −163 °C, there is still a considerable number of dimples in the core fracture, which proves that the material as a whole is far from reaching the tough–brittle transition temperature. There are carbides and ferrites in the P microstructure, so the P microstructure has a certain deformation ability and fracture toughness [62]. In the low-temperature environment, the P microstructure containing carbide will harden as a whole, and the hardness of the P will exceed the F. We speculate that the greater the hardness difference between P and F with the temperature decreases. The greater the stiffness, the more obvious the effect of the concentrated force. As a result, at a tensile temperature of −163 °C, a strong stress concentration occurs, so that the junction of F and P microstructure is pulled apart, forming an expandable crack, and there is an obvious fracture crack at the −163 °C core. It is important to note that the P microstructure is not completely brittle and non-deforming at −163 °C, as can be demonstrated by Figure 9a2,d2. The overall dislocation density of the core microstructure is less affected by the tensile temperature. At different tensile temperatures, there are subtle differences in the increment of new subgrain boundaries in the core microstructure, and the new subgrain boundaries tend to originate at precipitated phases, carbides, and grain boundaries [33,63]. The 25 °C specimen has more new subgrain boundaries, and the number of new subgrain boundaries decreases slightly as the temperature decreases. The more subgrain boundaries appear, the more deformation energy absorbed by the material deformation [33], corresponding to a tensile elongation of 26% at 25 °C and 16% at −163 °C.

The border TM microstructure is more homogeneous than the core F + P microstructure in terms of uniformity. We stretched the fracture and found no obvious fracture cracks. The deformation resistance of border TM microstructure is greatly affected by ultra-low temperatures. As shown in Figure 10, at a tensile temperature of −163 °C, the border microstructure is almost not deformed in the tensile direction, or the deformation along the tensile direction is small. The deformation degree of border microstructure can reach the level of core microstructure at 25 °C, but with the decrease in temperature, the deformation ability of TM decreases significantly, because the TM microstructure contains a higher proportion of carbonaceous precipitates than core F + P, and the effect of hardness at a low temperature is more obvious. Due to the effect of low temperatures, the number of new subgrain boundaries in the TM microstructure decreases dramatically. The overall dislocation density of the border TM microstructure is greatly affected by the tensile temperature and is the highest in the −163 °C sample. Because the deformation ability of it in the low-temperature environment is greatly reduced, the dislocation accumulation is obvious. The lower the tensile temperature, the more obvious the dislocation accumulation of the TM microstructure, and the strength of it is greatly improved in the low-temperature environment. The mechanism of strengthening is that it is more difficult to deform the TM microstructure at low temperatures, higher energy is required to make the matrix generate new dislocations, and the increment of dislocation strengthening in the strengthening mechanism is greatly increased [58], so that the tensile strength of −163 °C is 176 MPa higher than that of 25 °C, and the yield strength is increased by 100 MPa.

### 4.3. Advantages of Duplex Microstructure Design in Low-Temperature Deformation Environment

The low-temperature-resistant steel bar of 500 MPa steel bar is inseparable from the duplex structure design. The core F + P and border TM microstructure of low-temperature-resistant steel bars react differently in tensile environments from 25 °C to −163 °C. The core is guaranteed at −163 °C for low elongation, and the border gives the material a higher overall strength. Duplex microstructure fits, so as to ensure the low-temperature toughness and strength to meet the standard, which is one of the advantages of duplex structure design.

**Figure 19:**
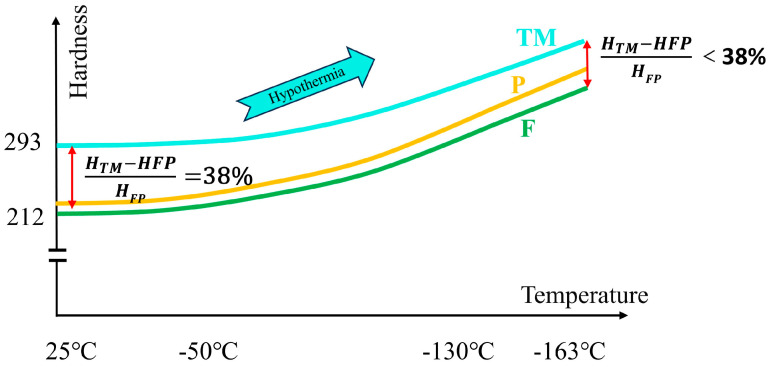
Trend of matrix microstructure hardness with ambient temperature.

As the ambient temperature decreases, the materials tend to become brittle and hard [60]. At room temperature, the HV_0.3_ of the heart is 212, and the HV_0.3_ of the edge is 293. The hardness of the edge is 38% higher than that of the heart. We propose a new concept here. Combined with the tensile deformation mechanism and microscopic analysis of low-temperature-resistant steel bars, the low-temperature environment will make the microstructure in the steel bar harder as a whole, with the largest increase in F, followed by the P structure, and the smallest in TM. The trend of microstructure hardness following temperature is shown in Figure 19. The implication of this concept is that the cold environment will cause the microstructure to harden, but the lower the temperature, the relative difference between the stiffness of the border and the core will decrease. According to the relationship between hardness and force distribution, on the whole, the low-temperature environment is conducive to the overall synergistic force of the core and border materials and slows down the stress concentration effect of the border materials.

### 4.4. Performance Comparison

**Figure 20:**
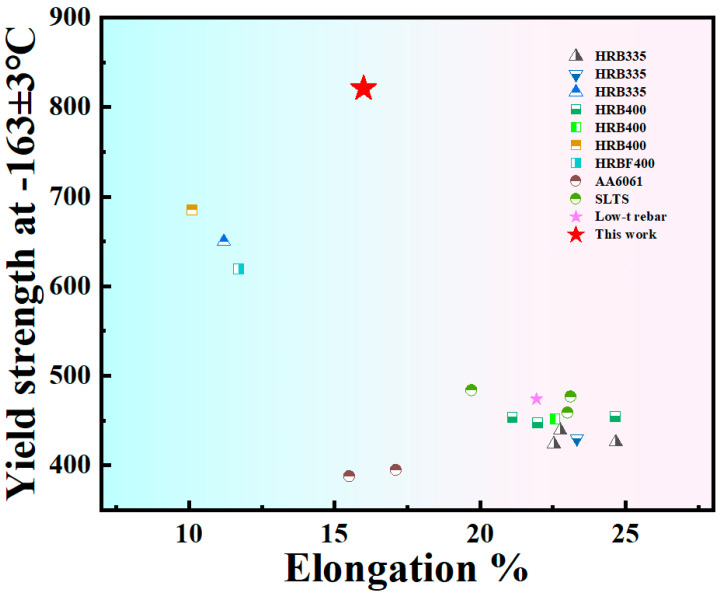
Comparison of performance of low-temperature-resistant steels at −163 °C ± 2 °C [10,23,64,65].

Previously, some scholars did a low-temperature tensile test of steel bars at −163 °C ± 2 °C [10,23,64,65,66], and the steel studied by these scholars was either low in strength or low in elongation, which we summarize in Figure 20. In order to meet the market’s demand for low-temperature strength and toughness of new steels, we designed 500 MPa steel grade low-temperature-resistant steel bars, which can ensure relatively high yield strength and maintain a certain elongation in an ultra-low-temperature environment of −163 °C. We expect that, in our next work, by reducing the thickness of the TM ring, the elongation at ultra-low temperatures will be further improved.

## 5. Conclusions

In this paper, a low-temperature-resistant steel bar with duplex microstructure of 500 MPa steel grade was developed and designed and has achieved industrial production. It adopts a continuous water-penetrating rolling process and self-tempering process to effectively control the microstructure and proportion of steel bars at room temperature, effectively cope with ultra-low-temperature tensile failure at −163 °C, and meet the performance requirements of 500 MPa steel grade low-temperature-resistant steel bar, which has a wide range of application scenarios. The material has good low-temperature resistance, which can meet the construction needs of high-strength and low-temperature-resistant steel bars of LNG storage tanks.

(1) The lower the tensile temperature, the higher the yield strength and tensile strength of the low-temperature-resistant steel bar, and the lower the plasticity. In the low-temperature environment, the tensile strength is increased, and the dislocation enhancement is dominant. The yield strength at 25 °C tensile temperature is 621 MPa, the tensile strength is 697 MPa, and the elongation after fracture is 26%. The yield strength at −163 °C is 821 MPa, the tensile strength is 873 MPa, and the elongation after fracture is 16%. The real performance exceeds the set standard, and there is a considerable margin of strength. The strain-hardening rate increases as the temperature decreases.
(2) The deformation behavior of the F + P microstructure in the core is less affected by the decrease in temperature, and the deformation behavior of the TM microstructure in the border is more affected by the decrease in temperature. At the tensile ambient temperature of −163 °C, the core microstructure still has a good deformation ability, and the border microstructure has only a slight deformation. The core microstructure ensures the 16% elongation of the low-temperature-resistant steel bar under ultra-low-temperature tensile. The tensile strength is greatly improved, and the border microstructure makes the greatest contribution.
(3) The uniformity of the material is an important reference in the low-temperature environment. A large number of cracks appeared at the fracture of the core of the low-temperature-resistant steel bar at −163 °C. Because there are two kinds of microstructure (F + P) in the core, the uniformity is not as good as that of the border microstructure. The core material has about 5% P microstructure, and we observe that the border of the deformed P microstructure is easily accompanied by microcracks and cavities, which is due to the stress concentration phenomenon at it, which leads to the separation of microstructure P and F. The lower the temperature, the more obvious the stress concentration effect, and the microstructure P is the root cause of the crack in the core of the rebar in the ultra-low-temperature tensile environment.
(4) The 500 MPa low-temperature-resistant steel bar is fully up to standard, and the yield strength even has a large margin. For the LNG storage tank temperature of −163 °C, our suggestion for improvement is to reduce the thickness of the border group TM ring a bit, which is now 2.6 mm and can be controlled at 2.0–2.2 mm. In the use scenario of −163 °C, the yield strength still reaches the standard, the strong yield ratio will increase, and the elongation will be further improved.

## Figures and Tables

**Table 1:** Chemical composition table of low-temperature-resistant steel bar.

Steel	C	Si	Mn	Ni	V	Ti	P	S	Ca	Al	N/ppm	Fe
HRB500DW	0.07	0.30	1.51	1.03	0.08	0.005	0.007	0.002	0.0025	0.0278	0.0081	Bal.

**Table 2:** Summary of preliminary characterization results.

Steel	Location	Metallograph	HV_0.3_	Grain Size
HRB500DW	Core	F + P	212	4.76
	Border	TM	293	6.05

**Table 3:** Test parameters of forward and reverse bending.

Steel	Test	Nominal Diameter	Diameter of the Bending Indenter	Results
HRB500DW	Positive bend	≤16 mm	4 d	Qualified
		>16 mm	7 d	Qualified
	Reverse bend	≤16 mm	5 d	Qualified
		>16 mm	8 d	Qualified

**Table 4:** Requirements for mechanical properties of 500 MPa steel grade with low temperature resistance and normal temperature.

Steel	R_eL_/MPa	R_m_/R_eL_	A_gt_/%
HRB500DW	500–650	≥1.10	≥5.0

**Table 5:** Requirements for low-temperature mechanical properties of 500 MPa steel grade low-temperature-resistant steel bar.

Steel	R_eL_/MPa	A_gt_/%	Specimen Style
HRB500DW	≥575	≥3.0	No notched
	-	1.0	Notched

**Table 6:** Results of low-temperature axial tensile test of low-temperature-resistant steel bar.

Steel	Diameter (mm)	Tensile Temperature	R_m_/MPa	R_eL_/MPa	A%	A_gt_%
HRB500DW	20	25 °C	697	621	26	11.06
HRB500DW	20	−50 °C	732	643	21	10.50
HRB500DW	20	−130 °C	799	704	20	10.00
HRB500DW	20	−163 °C	873	821	16	8.64

**Table 7:** Statistics of grain parameters of low-temperature-resistant steel bar before and after stretching.

Sampling Location	Specimen	Grain Block Size/μm	Grain Boundary ≥15°	Grain Boundary ≤15°	Kernel Average Misorientation				
					0–1.0	1.0–2.0	2.0–3.0	3.0–4.0	4.0–5.0
Core	Matrix	3.76	68.7	31.3	93.8	5.3	0.8	0.1	0
	25 °C	1.94	33.1	66.9	23.5	37.8	28.4	8.7	1.6
	−50 °C	2.27	26.8	73.2	20.3	37.0	31.0	10.1	1.6
	−130 °C	2.17	26.9	73.1	18.9	36.8	31.6	10.9	1.7
	−163 °C	2.18	26.6	73.4	18.7	35.4	32.9	11.3	1.9
Border	Matrix	3.37	33.1	66.9	38.4	46.4	12.7	2.1	0.4
	25 °C	1.95	24.5	75.5	24.5	36.5	28.2	6.4	0.9
	−50 °C	2.20	28.9	71.1	20.4	43.6	28.5	6.7	0.8
	−130 °C	2.61	23.1	76.9	21.6	41.1	28.8	7.4	1.1
	−163 °C	2.97	25.4	74.6	19.7	45.0	28.0	9.1	1.7

## Data Availability

The original contributions presented in this study are included in the article. Further inquiries can be directed to the corresponding authors.
